# How Does Migration Working Experience Change Farmers’ Social Capital in Rural China?

**DOI:** 10.3390/ijerph192013435

**Published:** 2022-10-18

**Authors:** Liang Chi

**Affiliations:** Agricultural Information Institute, Chinese Academy of Agricultural Sciences, Beijing 100081, China; chiliang@caas.cn

**Keywords:** migration experience, social capital, risk attitude, ICTs

## Abstract

While a growing number of farmers migrate to urban sectors to engage in off-farm employment, little is known whether and how the migration working experience (MWE) changes farmers, especially their social capital. Using a survey data set with 2863 farm households in 14 provinces in China, we developed a mediation model to examine the impact of MWE on social capital, as well as the roles of household income, farmers’ risk attitude, and information and communications technologies (ICTs). We show that MWE has a significantly positive impact on social capital and weak ties in social capital, which is mediated by household income, risk attitude, and ICT adoption. In particular, MWE can increase income, enhance risk preference, and promote ICT adoption, thus, leading to higher social capital. Moreover, ICTs play a moderating role in the impact of MWE on income and risk preference, that is, ICTs can decrease the impact of MWE on income, and completely substitute the impact of MWE on risk attitude. Our study provides an explanation for the reason why farmers are willing to migrate despite unfavorable working conditions they may endure in urban areas.

## 1. Introduction

Over the past century, with urbanization and industrialization, the number of migrant workers has been increasing around the world. The amount of migrant workers has reached about 250 million, according to available estimates, which would continue to increase in the future. However, various studies have shown that the welfare of migrant workers has been faced with challenges, such as mental problems, discrimination, and unfavorable social security [1,2]. The group of migrant workers has raised a lot of concerns.

In fact, the number of migrant workers has been underestimated. Due to China’s special system of residential registration (Hukou), a large number of farmers with the characteristics of migrant workers are not included in the statistics, and they face the same dilemma as international migrant workers. According to the official statistics from the Chinese government, there are 292.51 million migrant workers in China in 2021. Of the 292.51 million migrant workers, only 133.09 million of them have fixed residences in urban areas [3], while more than half of the migrant workers live in rural villages most of the time. In addition, because of the registered residence system and other unique policy factors in China, the group of migrant workers have to engage in heavy physical and intensive work, and they are faced with hard work and living situations. For instance, they are involved with “3D” jobs that are dangerous, dirty and demanding; they are exposed to poor working conditions; they have limited access to the same welfare of the healthcare system, education, and housing, compared with the local residents [4,5,6,7,8,9].

A question arises; faced with such poor working conditions and social welfare, why are these farmers willing to travel thousands of miles from home to engage in migration work? Whether and how the migration working experience changes them? We believe that the traditional economic analysis which mainly focuses on the pursuit of economic returns can no longer reasonably explain these phenomena. In this study, we provide a robust estimation of the effects of migration working experience on a farmer’s social capital, as well as the influence mechanism in rural China.

The objectives of this study are two-fold. The first is to apply a mediation model to explore how migration experience affects social capital through risk attitude, household income, and ICT adoption. The second is to investigate how ICT adoption mitigates the effects of migration experience on social capital through changing risk attitudes and household income. Specifically, migration experience changes the farmers’ risk attitude, increases household income and promotes ICT adoption, resulting in increasing farmers’ social capital. Meanwhile, the adoption of ICTs has a substitution effect on the migration experience affecting farmers’ risk attitude and household income.

To our knowledge, this study is among the first to investigate the effects of migration experience on farmers’ social capital, along with its mechanism in rural China, and therefore, help shed light on the issue. What is more important is that our study also has important implications for developing countries with similar characteristics of massive rural-to-urban labor migration to China.

The remainder of this paper is organized as follows. In Section 2 we provide a theoretical analysis and research hypothesis, followed by the materials and methods in Section 3. Section 4 presents the estimation results. Section 5 presents a discussion, and Section 6 concludes.

## 2. Theoretical Analysis and Research Hypothesis

The existing studies have investigated the migrant workers’ health conditions, and show that migrant workers endure worse mental conditions compared to local workers [10]. Similar evidence was found with internal migrants in China [11,12,13]. For example, a study examined the prevalence and the socio-demographic correlations of the mental health of migrant workers in Shanghai China and found that 25% of the male and 6% of female migrant workers could be classified as mentally unhealthy [14].

China has implemented the system of residential registration (Hukou) since 1958. Under this institutional arrangement, each citizen can be categorized as having an “urban” or “rural” belonging [12]. Due to the implementation of the Hukou system, public resources and social welfare are unevenly distributed between rural and urban areas [15]. In other words, urban citizens with a local Hukou enjoy better services than those who do not have Hukou. Consequently, rural-to-urban migrants do not have access to full citizenship rights and are unable to enjoy the same social, economic, and political rights [16,17,18], and are often treated as “second-class citizens”, suffering from discrimination in housing, education, employment, and health care in cities [19].

Despite migrant workers being confronted with these horrible situations, still, Chinese migrant workers have seen a steady rise since rural reforms and open policies were implemented in China from 1979, with 292.51 million rural migrants in 2021 [20]. Some studies suggest that since migrants typically leave their homes to improve their economic status and increase job opportunities, this may lead to improved psychological health [21,22]. In particular, China is a country with the largest population and amount of urban-to-rural migrant workers; it is necessary to pay attention to the social welfare of this group of people. 

For most migrant workers, they migrate to work to find a stable employment opportunity and acquire the Hukou in urban cities, since they would be able to enjoy better public services, including education, housing, health care, and social security. Historically, very few migrant workers made it and stayed, while others would have to move periodically between the city and the countryside. For those people, the most typical and largest group of Chinese migrant workers, what has the migration working experience changed for them?

Several questions arise, why do these migrant workers, namely farmers, choose to migrate to urban areas? What impact it has on migrants? How it changes farmers? Most of the existing studies are focused on the physical and mental health and well-being of migrant workers, with little knowledge on whether and how the migrant working experience changes the farmers, especially their social capital. Maslow’s demand theory provides a possible theoretical explanation for the behavior of Chinese migrant workers. They know that there is little chance of them settling down in the cities for migrant work, but they still prefer to sacrifice their health and welfare and move between the cities and the countryside in cycles. Do migrant workers have other pursuits beyond economic returns and social welfare, like social approval? 

Although there have been several studies investigating the impact of migration on social capital, the results are unclear or even conflicting. Some literature suggest that the increasing migrant population in agricultural fields augments their social isolation because the growing competition for scarce employment opportunities dissolves social and relation bonds [23], others argue that migration experience contributes to promoting social capital [24]. Moreover, the influence mechanism remains unclear and has not yet been fully understood.

Our study may contribute to explaining why a large number of farmers are still willing to engage in migration work even though they may suffer from poor working conditions and social welfare. More importantly, we have not only shown that this effect exists, but we have also shown some possible mechanisms of influence. In the process of analyzing the results of the economic model, we introduce representative cases to further prove our conclusion. Through the review of previous studies, we found the following possible influencing mechanisms, and on this basis, we proposed a research hypothesis.

In general migrant workers are faced with two options, either stay and keep seeking better life prospects in cities or go back to their hometowns. For those who decide to come back, they are likely to have higher social capital than those local farmers who don’t have migrant working experience. Firstly, the migration experience has a positive impact on labor income [25], and the returnees earn more than the overall wage earners with the same education and skill levels [26], and have better chances of becoming entrepreneurs [24,27]. That is, farmers with migration experience tend to make more money and become wealthier than their local counterparts, considering the wage differences between the non-agricultural sectors and agricultural sectors, and thus, enrich social capital.

Meanwhile, farmers with migration experience are likely to prefer risks and be more adventurous, since migration can be seen as a kind of risk investment, and risk attitudes can produce an effect on the migration decision [28,29]. The rural-to-urban migrants and their family members are substantially less risk-averse than stayers [30]. The risk attitude, in turn, may have an impact on their social capital, since risk aversion is strongly correlated to local network clustering, that is, the probability that one has a social tie to friends of friends [31]. Particularly, in our study, risk attitude is defined as the traditional concept of economics. Risk attitude refers to people’s attitudes towards risk. Specifically, it is a mental state when people are faced with positive or negative uncertainty. In other words, it is the way people choose to respond to perceptions of important uncertainty. Risk attitudes are generally divided into three types: Risk averse, Risk neutral, and Risk appetite.

More importantly, the development of information and communications technologies (ICTs) has reshaped people’s lives and behaviors. ICTs provide easier access to social support [32], multiplex stronger social network structures [33], and more complicated personal relationships [34,35]. These social networks are the fundamental resources for them to create social capital [36], especially for migrant workers [37]. Hence, farmers with migration experience are likely to access more ICTs, and thus, have higher social capital than those who do not.

**Figure 1:**
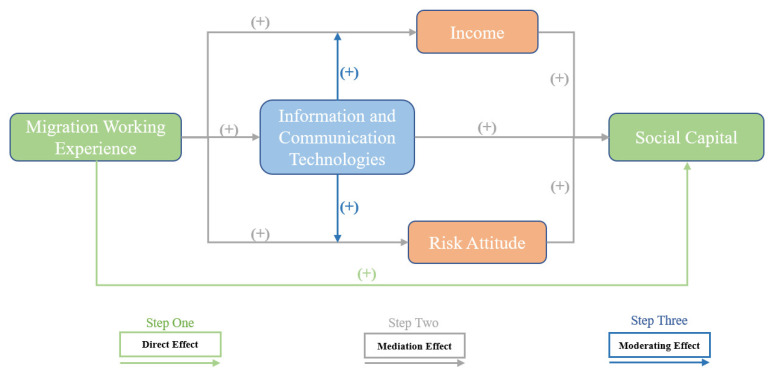
The influence mechanism of MWE on SC.

Based on our observation and previous studies, we established a conceptual framework, considering the role of income, risk attitude, and ICT adoption in the effects of farmers’ migration working experience on their social capital. A possible mechanism is shown in Figure 1. Hence, we propose the following hypothesis:

**H1.**  *Migration experience has a significantly positive effect on farmers’ social capital.*

**H2.**  *The impact of migration experience on social capital is positively mediated by risk preference.*

**H3.**  *The impact of migration experience on social capital is positively mediated by household income.*

**H4.**  *The impact of migration experience on social capital is positively mediated by the adoption of information and communications technologies (ICTs).*

**H5.**  *ICT adoption has a moderating effect, and can help mitigate the impact of migration experience on household income and farmer risk attitude.*

## 3. Materials and Methods

### 3.1. Empirical Models

#### 3.1.1. Mediation Model for Baseline Regression

In order to examine the mechanism of how the migration working experience (MWE) affects farmers’ social capital (SC), we employ a mediation model to explore whether farmers’ income, risk attitude, and ICT adoption mediate the effect of MWE on SC. The mediating effect mainly tests the role of household income, farmer risk attitude, and ICT adoption. The three-model system is widely used and constructed to examine the mediating effects of mediators, we set up the three-model system as follows:

(1) $$SC_{i}=\gamma_{0}+\gamma_{1}MWE_{i}+\gamma_{2}X_{ki}+\varepsilon_{1i}$$

(2) $$M_{i}=a_{0}+a_{1}MWE_{i}+a_{2}X_{ki}+\varepsilon_{2i}$$

(3) $$SC_{i}=\rho_{0}+\rho_{1}MWE_{i}+\rho_{2}M_{li}+\rho_{3}X_{ki}+\varepsilon_{3i}$$

Here, $MWE_{i}$ indicates the migration working experience of the farmer i; $M_{i}$ is income, risk attitude, and ICT adoption of the farm i, namely, the mediators; $X_{ki}$ is a vector of other variables affecting SC such as the farmer’s education level and $\varepsilon_{i}$ is a random error term.

Specifically, we first test the direct effects of MWE on SC without considering the three mediator variables in Equation (1). Then, we explore the effects of MWE on three mediator variables in Equation (2). The last step is to investigate the effects of MWE and three mediator variables on SC in Equation (3). If we find $a_{1}$ equal to 0, $\rho_{2}$ equal to 0, or $\rho_{1}$ equal to $\gamma_{1}$, then we cannot reject the null hypothesis that there is not a mediating effect.

#### 3.1.2. Moderating Effects of ICT Adoption

To better understand the role of ICT adoption in the relationship among MWE, income, risk attitude, and SC, we introduce a dummy variable, ICT adoption. Both this dummy variable and its interaction with MWE are incorporated into the regression, so that:

(4) $$Income_{i}=m_{1}+m_{2}MWE_{i}+m_{3}X_{ki}+m_{4}ICT_{i}+m_{5}MWE_{i}\times ICT_{i}+\varepsilon_{4i}$$

(5) $$Risk\;attitude_{i}=n_{1}+n_{2}MWE_{i}+n_{3}X_{ki}+n_{4}ICT_{i}+n_{5}MWE_{i}\times ICT_{i}+\varepsilon_{5i}$$

### 3.2. Data

This study utilizes a data set which was obtained by a face-to-face questionnaire survey administered by the National Agricultural and Rural Development Research Institute (NARI) of China Agricultural University (CAU) in 2019. Multistage sampling was employed for data collection. First, 14 provinces were chosen. Second, the towns were selected in each province based on the grain production area. Then, 1–2 villages were randomly selected from each town. Next, 15–20 farm households were chosen from each village.

**Figure 2:**
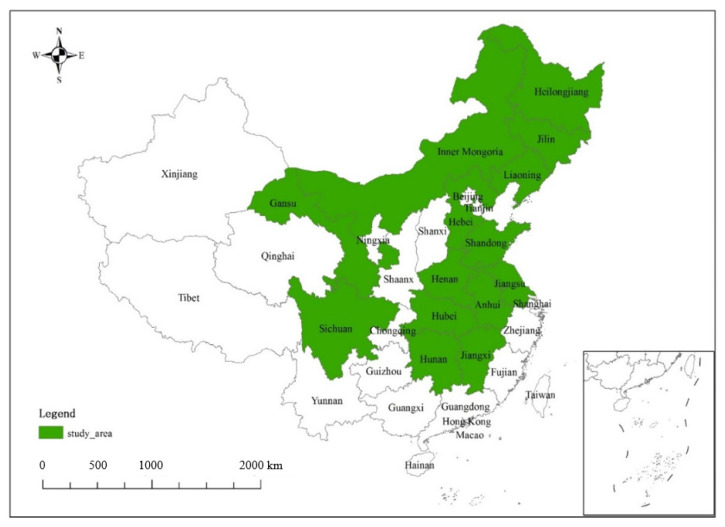
Profile of study areas.

High-quality survey data is one of the most important advantages of our survey. From November to December 2018, the NARI recruited the most qualified interviewers, mostly students from CAU, and trained them to guarantee that they can collect appropriate data during the survey. In fact, most of the students already had sufficient experience in surveys. The survey was conducted from January to February 2019 when the university was on winter vacation. After cleansing and dropping the inconsistent and incomplete questionnaires, the final data set consists of 2863 farm households across 14 provinces, namely Inner Mongolia, Jilin, Sichuan, Anhui, Shandong, Jiangsu, Jiangxi, Hebei, Henan, Hubei, Hunan, Gansu, Liaoning, and Heilongjiang, as is shown in Figure 2. 

Observations from the 14 provinces are shown in Table 1. 

### 3.3. Variables

**Dependent variable**

In our study, we focus on the impact of the farmer’s migration working experience on social capital and its influence mechanism. Our dependent variable is social capital (SC), which is measured as two indicators.

**Social capital**. The Spring Festival, namely Chinese New Year, is the most important traditional holiday in China. During the Spring Festival, people would visit and greet each other. The higher a person’s social status is, the more people would come and visit. With the popularization of Internet technology, people would greet each other via phone calls, instant messaging software, and other modern technologies during the Spring Festival. Although the ways that people greet each other have changed, what’s underneath remains, that is, more greetings mean stronger social capital. In our study, social capital is measured as the amount of people that the farm household received greetings from in various ways, including video calls, phone calls, WeChat, etc., during the Spring Festival.

**Weak ties in social capital.** The structure of social capital is complex and includes family members, relatives, people in the same villages, and ordinary friends. Social relations can be categorized into two groups, strong ties and weak ties. In particular, strong ties denote social relations formed naturally and people don’t have to nurture them intentionally, including relations with family members, relatives, and people in the same villages. Weak ties represent social relations that the farmer has to nurture and maintain, in our case, with ordinary friends. In our study, we measure weak ties in social capital using the percentage of the number of greetings received from ordinary friends in the total number of greetings.

It is important to observe the differences between strong ties and weak ties, especially in rural China, with the special influence of village relationships in traditional culture [38]. We take into account weak ties since kinship and natural factors are excluded from weak ties in social capital. In this way, we can capture the actual social capital increased by MWE more accurately.

**Independent variables of interest**

We use two variables to measure migration working experience, MWE and MWE-year. The former is used for baseline regression, the latter is for robustness test.

**MWE**. We use a dummy variable, whether the farmer has once engaged off-farm employment, to measure MWE. In our questionnaire, farmers were asked about the specific place they worked as migrant workers. At the same time, we gave a very specific definition of the distance of migration, which can be divided into four levels: outside village, outside township, outside county, and outside province. 

Since the differences between neighboring villages within the same town in China are relatively small, and people are most likely to engage in agricultural production in the village, we only consider the migration experience outside the town. That is to say, the experience of a farmer who migrated to the neighborhood village is not considered to have MWE. 

**MWE-year**. To provide robustness checks, we use the years that the farmer engaged in off-farm employment (the same definition as mentioned above, but only those more than six months in a single-year count), to replace MWE dummy variable and rerun the regressions.

**Mediators**

**Income.** It is measured as the total income of the farm household in 2018, including agricultural income and non-agricultural income.

**Risk attitude.** Particularly, in our study, risk attitude is defined as the traditional concept of economics. Risk attitude refers to people’s attitudes towards risk. Specifically, it is a mental state when people are faced with positive or negative uncertainty. Or, in other words, the way people choose to respond to perceptions of important uncertainty. Risk attitudes are generally divided into three types: Risk averse, Risk neutral, and Risk appetite. 

In the survey process, we used the research methods of the most authoritative survey databases in China, such as the China Household Finance Survey (CHFS), to measure the risk attitude of farmers. The survey asked the farm household head a question, “if you have 10,000 CNY to conduct financial investment, which of the following three choices do you prefer?” Choice 1 = “earn 400 CNY (4%) in the best case and no loss in the worst case”, 2 = “earn 1700 CNY (17%) in the best case and loss 1000 CNY (10%) in the worst case”, and 3 = “earn 9600 CNY (96%) in the best case and loss 4800 CNY (48%) in the worst case”. A larger value means a higher risk preference. 

**Information and communications technologies (ICTs)**. We use a dummy variable, whether the farmer used a smart phone or personal computer to search for information over 4G mobile networks or the Internet, or connect people by an instant messaging software such as WeChat, to measure ICT adoption. We take WeChat as an important indicator of information technology adoption because WeChat is an important way for farmers in rural China to obtain information. WeChat integrates the RSS function and search function, so farmers can not only receive an information push, but also take the initiative to obtain information.

**Control variables**

Following the existing studies, we control for farm household and farmer characteristics, including the age, gender, education, and health condition of the farm household decision maker, village cadres experience, labor allocation, hilly land ratio, agricultural income ratio, etc. [39,40,41,42,43,44,45,46].

### 3.4. Descriptive Statistics

A statistical description of variables is presented in Table 2. It shows that 41.4% of the farm households had off-farm work experience over 6 months, while the rest of them engaged in agricultural production most of the time. Meanwhile, the majority of farmers chose a conservative financial investment strategy. The social capital shows significant differences among the farm households, as the standard deviation is greater than the mean and the extreme value is large. The weak ties in social capital (a proxy of social capital structure) are small, which means that most of the farm households’ social relations are still focused on their families or closer friends, that is, strong ties. 

The average age of the farm household head is over 50 years old. The average education level is primary school and junior-middle school, implying that the human capital of rural households is relatively small. The self-reported health condition is good, and the proportion of village cadres is low, reflecting the realistic constraints of the relatively low level of human capital of the interviewed farmers. Most of the household labor force is still concentrated on agricultural production, but the source of income of most families does not depend on agriculture, reflecting the limited contribution of agriculture to farmers’ income.

By comparing the surveyed farmers with and without MWE, as shown in Table 3, it can be found that there are significant mean differences in social capital and weak ties in social capital between the two groups: farmers with MWE have a larger number of people who connect with them during the Spring Festival. More importantly, farmers in MWE groups have higher weak ties in social capital. A lower proportion of the number of family members, relatives and local residents means they have a wider range of social contacts. This means that farmers in the MWE group have the opportunity to enhance the ability of information acquisition and factors acquisition, and improve the marginal return of factors with the role of higher social capital.

## 4. Results

### 4.1. Baseline Regression

We examine the effect of MWE on SC in three steps. Firstly, we calculate the direct effect of MWE on SC. Then, analyze the mediating effect of farmers’ characteristics change, that is, how the MWE affects farmers’ income and risk attitude, and then affects their social capital. Finally, we talk about the influence of ICTs, on the one side ICTs have a mediation effect between MWE and SC, on the other side, ICTs also have a moderating effect on the effect of MWE on farmers’ income and risk attitude.

#### 4.1.1. Direct Effects of MWE on SC

Table 4 reports the direct effect of MWE on SC. It shows that, without considering the influence of other factors, MWE can directly increase farmers’ social capital as shown in column (1). This result is consistent with what our research team learned during the survey. The positive impact of migrant work experience (MWE) on farmers’ social capital is mainly reflected in two aspects.

On one hand, it is clear that leaving rural areas to work in different places will provide farmers with more opportunities to make new friends, and thus, increase their social capital. An interesting phenomenon can be found in our survey, that is, the social connections of migrant workers in cities can be divided into two obvious groups. One social group is contacts from work, the other is fellow townsmen, i.e., people who work in the same city and came from the same hometown. It should be noted that fellow townsmen play an important role in the social relations among migrant workers and the friendship associations among them are extremely tight. This phenomenon is inseparable from China’s traditional culture for thousands of years, just like the old Chinese saying that tears swim in the eyes when fellow townsman meet each other. Establishing social networks through fellow villagers’ friendly feelings is also an efficient way for farmers to quickly adapt to the new working environment in cities.

On the other hand, migration work also increases the scope of connections between farmers and their village residents and relatives. Firstly, their friends and relatives in hometowns tend to consult them in terms of agricultural production decision making, agricultural technology adoption, and even children’s education. Working in the city means broader insight and richer sources of information, which can help them make more convincing and rational decisions. Secondly, consistent with the findings of previous studies, farmers who work in cities are likely to reach out to their friends and acquaintances from their hometown through social networks. The longer the farmer stays in the city and more familiar with the city, they are likely to be contacted by their friends and acquaintances from their hometown.

To sum up, the empirical analysis based on the survey data at the national level shows that MWE can significantly increase farmers’ social capital. The survey data also shows that the increase in farmers’ social capital mainly has two sources: one is new friends made in the city due to their work, and the other is people who are still in rural areas but expect to obtain relevant information or job opportunities through them. 

#### 4.1.2. Mediating Effects

Our results show that MWE can increase social capital, but little is known about the influence mechanism. Here, we imply a mediation model to analyze the role of income, risk attitude and ICT adoption in the effect of MWE on SC. The estimated results are shown in Table 4. 

It shows that the coefficient of MWE on SC is significant and positive in columns (1), (3), (5), and (7), implying that MWE has a significantly positive effect on SC. The coefficient of MWE on farmers’ income, risk attitude, and ICT adoption is significant and positive, in columns (2), (4), and (6), respectively, which means that IEW has a positive impact on farmers’ income, risk attitude, and ICT adoption. Meanwhile, the coefficient of farmers’ income, risk attitude, and ICT adoption on SC are significantly positive in columns (3), (5), and (7), respectively, meaning that they have significantly increased farmers’ SC. The results suggest the existence of the mediating effect of income, risk attitude, and ICT adoption, and the total effect mediated by them is 21%, 16%, and 31%, respectively. As expected, migration working experience can not only directly increase the SC of farmers, but also indirectly increase the SC by increasing the farmers’ income, changing their risk attitude, and promoting ICT adoption.

#### 4.1.3. Moderating Effects of ICT Adoption

To examine the effect of ICT adoption on the relationship between MWE and farmers’ income, we apply OLS regression on Equations (4) and (5), the results are shown in Table 5. Without considering the impact of ICT adoption, MWE has a significantly positive effect on farmers’ income as shown in column (1). After ICT and the interaction term of ICT adoption and MWE were introduced into the regression, we can see from column (2) that ICT has significant and positive coefficients, implying that ICT adoption can significantly increase farmers’ income, but the interaction term of MWE and ICT has significant and negative coefficients, implying that there is a substitution effect between MWE and ICT adoption on the effect of farmers’ income. 

The effect of ICT adoption on the relationship between MWE and farmers’ risk attitude is obviously different from income, we also apply OLS regression on Equations (4) and (5), the results are shown in Table 5. The direct effect of MWE on farmers’ risk attitude is significant and positive as shown in column (3), then we introduce ICT and the interaction term of ICT adoption and MWE into the equation, they both have significant and positive effects on farmers’ risk attitude, but the coefficient of MWE changes from significant to insignificant, and the coefficient also becomes negative, what implies that MWE can increase farmers’ risk preference, but ICT adoption is the core explanatory variable affecting farmers’ risk attitude compared with MWE.

### 4.2. The Effects of MWE on Social Capital Structure

**Figure 3:**
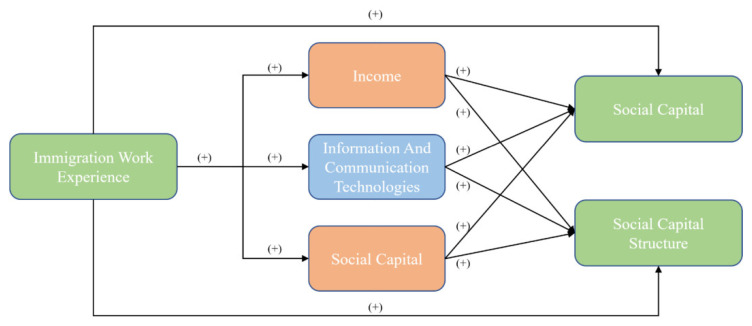
Influence Mechanism of MWE on Social Capital Structure.

Based on the above theoretical analysis, the influence mechanism of migration working experience on social capital structure is shown in Figure 3.

In order to better understand how MWE affects farmers’ social capital, we take weak ties in social capital as an alternative explanatory variable to further analyze the impact of MWE and its impact mechanism.

Table 6 reports the direct effect of MWE on farmers’ weak ties in social capital (social capital structure). It shows that, without considering the influence of other factors, MWE can directly increase farmers’ weak ties in social capital as shown in column (1). All mediators play the same roles on the effect of MWE on weak ties in social capital like the effect of MWE on SC, but the results of the Sobel test show that ICT adoption plays even more important role, it can explain 40% of the effect of MWE on weak ties in social capital.

### 4.3. Robustness Check

We use two methods to test the robustness of the baseline regression, one is that we use the year of migration working experience (MWE-year) as the explanatory variable; another is that we use the propensity score matching (PSM) technique to reassess the impact of MWE on SC and weak ties in social capital. 

#### 4.3.1. Alternative Variable for MWE

Table 7 reports the direct effect of MWE-year on farmers’ SC and weak ties in social capital, only when the immigration work time of farmers in a year exceeds half a year, we count it as one year of migration working experience. This is a further strict definition, which can more accurately measure the migration working experience of farmers. The coefficients of MWE-year in both columns (1) and (2) are significant and positive, it means that the more years the farmers engaged in immigration work the more social capital they have. Furthermore, MWE also significantly enhances the weak tiles in social capital, meaning that the longer they engaged in off-farm work, the more tight social capital structure they have.

#### 4.3.2. Alternative Empirical Model

Based on the results of OLS model, we show that MWE is significantly positively correlated with farmers’ SC and weak ties in SC, and the effects are mediated by household income, farmer risk attitude, and ICT adoption. To further examine the impact of MWE on farmers’ social capital, we apply a PSM technique.

In our study, we use the following different matching methods, the nearest neighbor matching, caliper (0.03) matching, kernel matching (default 0.06 bandwidth), and local linear regression matching methods, respectively, to estimate the average treatment effect on the treated (ATT) generated by MWE for samples in the common support domain, that is, the net effect of MWE on social capital.

The results of the balance test of covariates are shown in Table 8. The covariate deviation between the two groups of samples was distinguished. The results show that the covariates after PSM matching pass the balance test, which is helpful to the subsequent analysis of the social capital differences between farmers with MWE and farmers without MWE.

As reported in Table 9, the PSM estimation results show that MWE has a significant causal effect on SC, which is consistent with our baseline regression results.

## 5. Discussion

### 5.1. Role of Income

There is an idiom in China called “returning home with gold”, which means that when you leave your hometown to work and achieve certain achievements or accumulate certain wealth, you will be respected by the people in your hometown, this concept has been deeply rooted in Chinese culture for thousands of years. Therefore, the pursuit of higher income is the core pursuit for farmers to leave their hometowns to work. Industrial and agricultural scissors differences have existed for a long time since the founding of the people’s Republic of China. In 1952, China’s agricultural net output value accounted for 74.7% of the industrial and agricultural net output value, and 83.5% of the employed population was engaged in agricultural production and the cottage industry. In the following decades, the unified purchase and marketing policy and the urban-rural registered residence system further expanded this income gap, this kind of economic growth competition has not only caused the growth imbalance between urban and rural areas but also caused the regional growth imbalance between the eastern coast and the central and western regions [47]. 

With the advancement of urbanization in China, the differences between urban and rural are enlarging and show significant regional differences. Specifically, the urban-rural gap in the eastern region is decreasing, while the urban–rural gap in the central and western regions is expanding, leading to the emergence of massive migrant workers. Most of them migrate from the central and western regions of China to the eastern region for higher income. These huge differences in remuneration between agricultural sectors and industrial sectors in China are inseparable from the evolution of urban–rural relations since the implementation of rural reform and open-up policy in 1979. Therefore, based on this historical background, migration working means a higher probability of income growth, which can improve one’s prestige in the hometown. It is, therefore, easy to understand how MWE affects farmers’ social capital. 

### 5.2. Role of Risk Attitude

Previous studies have paid little attention to the impact of risk attitude on farmers’ social capital. In our study, we pay attention to the role of risk attitude in the relationship between MWE and SC. MWE brings farmers additional non-agricultural income, which significantly improves their ability to resist risks and increase their risk preference.

On one hand, farmers with a higher risk preference have a greater probability of adopting new agricultural technologies and are more willing to adopt new agricultural technologies methods. Agricultural production enables farmers to link with each other, since farmers tend to consult and learn from those who adopt new agricultural production technologies. There is a similar influence mechanism in the sales of agricultural products, for example, the decision to use new sales modes of agricultural products, such as e-commerce. Hence, a higher risk preference enables farmers to accumulate experience and knowledge, which can significantly affect the social capital of farmers. As such, MWE play a role by the mechanism, that is, MWE–Higher risk preference–Higher probability of technology adoption, accumulation of information, experience, and knowledge–More farmers followers–Increasing social capital.

On the other hand, a higher risk preference means more job opportunities. In the early stage of China’s urbanization, farmers are likely to migrate, which significantly increases income. However, with the continuous advancement of urbanization, this situation has changed, more and more farmers engage in migration work, but the homogeneity of these labor forces is rather high. That is to say, those farmers with higher risk preferences would have more information through more radical and frequent work replacement, which enables them to have stronger social networks in urban areas. As such, MWE plays a role by the mechanism: MWE–Higher risk preference–More frequent job hopping–Accumulation of information, experience, and skills–More farmers followers–Increasing social capital.

### 5.3. Role of ICT Adoption

Compared with income and risk attitude, the effect of ICTs on the relationship between MWE and SC is more complex. On one hand, it plays the same role as MWE and SC do. On the other hand, ICTs have significant and heterogeneous effects on income and risk attitude.

For the mechanism “MWE-ICTs–SC”, considering “MWE–ICTs”, there are significant differences between urban and rural work and life styles. Obviously, ICTs are more widely used in cities, and farmers have to increase the frequency and intensity of ICT adoption to meet the needs of work and life. Considering “ICTs–SC”, ICTs can break geographical restrictions and significantly reduce the cost of communication between people, they can communicate with family members or villagers by instant messaging software such as WeChat. What is more, functions like group chat and WeChat Moments (similar to Facebook Messenger and Instagram) can easily connect hundreds of people or even more.

For the mechanism “MWE-(ICTs)–Income”, MWE can directly promote income, while the use of ICTs can slow down the process of MWE on income. It implies that the use of ICTs can be a substitute for migration to increase household income. From the logic of how MWE and ICTs increase household income, the results are implausible. As analyzed before, MWE plays a significant role in accessing more information, experience and knowledge through migration work. MWE has a positive impact on household income. ICTs can do the same thing since it lowers the cost of accessing information. During the field surveys, we also found that ICTs can easily break the geographical limitations among farmers, which decreases communication costs. 

For the mechanism “MWE-(ICTs)-Risk attitude”, MWE can directly promote farmers’ risk preference, and ICT adoption can enhance the effect of MWE on risk attitude. More importantly, ICT adoption has a complete substitution effect on MWE. Based on the analysis above, although we have known the key role of ICTs, the results still exceed our expectations. To some extent, it implies that the information obtained through ICTs and migration work has the same effect on the change of farmers’ risk attitude. Another possible explanation is that information brought by ICTs can provide migrant farmers with more employment information. It raises their expectations for the future, and therefore, they have higher risk preferences.

### 5.4. Further Discussion of Social Capital Structure (Weak Ties)

Based on China’s special rural culture, we believe that the analysis of the social capital structure is a necessary extension of the discussion. Xiaotong Fei, the founder of Chinese sociology, proposed “the Pattern of Difference Sequence” of Chinese rural society. It vividly describes the interpersonal pattern of closeness in Chinese rural society, a rating-circle structure from inside to outside refers to family members, relatives, fellow villagers and friends, and it also means that the social relations expand from strong social ties to weak social ties. It likes a halo spreading on the water, extending from oneself, dividing closeness and distance according to the distance from oneself. In traditional Chinese culture, there is a natural trust between relatives and fellow villagers, this leads to heterogeneity in the social capital structure. Therefore, we use the concept of social capital structure to reinforce the effect of MWE on weak social ties. 

The results show that the effects of MWE on social capital are multifaceted, which not only strengthens the connection between farmers and strong social ties, but also weak social ties. About the effects on social capital structure, we also found an interesting case during our survey. Migrant workers may spend more time away from their hometowns, and they have to maintain social relations at work. Therefore, they do not have to spend much more time to maintain strong social ties than they would have to maintain weak ties.

## 6. Conclusions

In this study, based on a survey data set of 2863 farm households in 14 provinces in China, mediating effect model and moderating effect model are used to analyze the effect of migration working experience (MWE) on farmers’ social capital (SC). We show that MWE has a significant positive impact on SC, which is mediated by household income, farmer risk attitude, and ICT adoption. Moreover, ICT adoption can mitigate the process of MWE affecting income and risk attitude, and have heterogeneous substitution effects. To provide robustness checks, we used the months of migration working and weak ties in social capital to replace core explanatory variables and dependent variables, respectively, and the estimation results are consistent.

Our results clearly indicated that MWE can significantly enhance farmers’ social capital by increasing income, promoting risk preference, and ICT adoption. Meanwhile, MWE is likely to have a strong externality, that is, one connects frequently with people who have MWE and would benefit from their accumulation of information, technologies, skills and knowledge. It is the key to how MWE enhances farmers’ social capital. 

This study contributes to a better understanding of the phenomenon that people are willing to work as migrant workers even though they may suffer from unfair treatment and the loss of welfare. More importantly, this study has specific policy implications. It is necessary and beneficial to encourage migration, because it not only improves the welfare of migrants, but also helps improve the welfare of those who stay in close contact with them. MWE plays a similar role to “Xiangxian”, a traditional Chinese cultural concept which means people of great prestige in the village. Of course, policies should be carried out by the government to guarantee the legitimate rights and interests of migrant workers, as many researchers and policymakers have noticed.

The generalization of this study is subject to certain limitations. For example, limited by financial support the study is focused on migrant workers from the main provinces in China instead of national-level survey. Future studies would consider a wider coverage of provinces. Meanwhile, we focus on the group of farmers who live in the rural areas, instead of those who live the urban areas. It is unknown whether migration working experience has a significant impact on social capital for this group of migrant workers. Further studies may focus on the working conditions and the physical and psychological conditions of the group of migrant workers living in rural areas. 

## Figures and Tables

**Table 1:** Distribution of observations by province/autonomous region.

Province	N	Percentage of Observations (%)
Inner Mongolia	241	8.42
Jilin	150	5.24
Sichuan	245	8.56
Anhui	130	4.54
Shandong	410	14.32
Jiangsu	209	7.30
Jiangxi	158	5.52
Hebei	245	8.56
Henan	311	10.86
Hubei	197	6.88
Hunan	162	5.66
Gansu	118	4.12
Liaoning	106	3.70
Heilongjiang	181	6.32
Total	2863	100.00

**Table 2:** Definition of variable and sample summary statistics.

Variable	Definition	Mean	St. Dev
Dependent variable			
Social capital	Continuous variable, the amount of received greetings from friends and acquaintances of the farm during the Spring Festival	54.58	77.69
Weak ties in social capital (Social capital structure)	Continuous variable, the percentage of the amount of received greetings from ordinary friends in the total amount of greetings during the Spring Festival	0.48	0.20
**Independent variable**			
MWE	Dummy variable, “1” if the farmer has off-farm working experience, “0” otherwise	0.41	0.49
MWE-year	Continuous variable, years that the farmer engaged in off-farm employment (more than six months in a single year)	5.07	9.01
**Mediator variable**			
Income	Continuous variable, household income, measured as the total income of the household in 2018, in natural log (ln)	10.49	2.06
Risk attitude	Ordered variable, of the three options to conduct financial investment with 10,000 CNY, 1 = “earn 400 CNY (4%) in the best case and no loss in the worst case”, 2 = “earn 1700 CNY (17%) in the best case and loss 1000 CNY (10%) in the worst case”, 3 = “earn 9600 CNY (96%) in the best case and loss 4800 CNY (48%) in the worst case”	1.48	0.70
ICTs	Dummy variable, information and communications technologies, “1” if the farmer used smart phone or personal computer to search information and connect people by instant messaging software such as WeChat, “0” otherwise	0.31	0.47
**Control variable**			
Age	Continuous variable, age of the household head	52.73	11.25
Male	Dummy variable, “1” male, “0” female	0.76	0.42
Education	Ordered variable, education level of the household head (1–6), Ordered variable, “1” illiterate, “2” elementary school, “3” middle school, “4” high school or vocational high school, “5” three-year college, and “6” college or post-graduate	2.76	0.95
Health	Ordered variable, “1” if the household decision maker’s health condition is great; “2” fine; “3” bad; “4” disabled	1.42	0.63
Village cadres	Dummy variable, “1” if farm household have the experience of village cadres	0.16	0.37
Labor allocation	Continuous variable, the percentage of off-farm employed labor in the total labor	0.30	0.27
Hilly land ratio	Continuous variable, the percentage of hilly land in the total operated land area (%)	0.09	0.23
Agricultural income ratio	Continuous variable, the percentage of agricultural income in the total income (%)	0.35	0.36
East	Dummy variable, “1” if farm household is located in eastern region, “0” otherwise	0.34	0.47
Central	Dummy variable, “1” if farm household is located in central region, “0” otherwise	0.53	0.50
West	Dummy variable, “1” if farm household is located in western region, “0” otherwise	0.13	0.33

**Table 3:** The differences between sample groups with and without off-farm work experience.

Variable	MWE	No-MWE	Differences	T Value
Social capital	60.161	50.855	9.306	3.1297 ***
Weak ties in social capital	0.502	0.474	0.028	3.5568 ***

Note: Standard errors are in parenthesis, *** *p* < 0.01.

**Table 4:** Direct Effects and Mediating Effects.

Var	(1)	(2)	(3)	(4)	(5)	(6)	(7)
	SC	Income	SC	Risk	SC	ICT	SC
Income			0.884 ***				
			(0.246)				
Risk					3.674 ***		
					(0.650)		
ICT							9.711 ***
							(0.953)
MWE	2.901 **	0.677 ***	2.303 *	0.125 ***	2.442 **	0.092 ***	2.007 *
	(1.188)	(0.103)	(1.194)	(0.034)	(1.185)	(0.023)	(1.171)
Control	Yes	Yes	Yes	Yes	Yes	Yes	Yes
_cons	35.333 ***	11.811 ***	24.895 ***	2.193 ***	27.276 ***	0.721 ***	28.334 ***
	(3.332)	(0.288)	(4.188)	(0.095)	(3.607)	(0.064)	(3.344)
Obs.	2863	2863	2863	2863	2863	2863	2863
Sobel Tests		0.598 ***		0.459 ***		0.894 ***	
		(0.172)		(0.149)		(0.239)	
Total effect mediated		21%		16%		31%	

Note: Robust errors are in parenthesis, *** *p* < 0.01, ** *p* < 0.05, * *p* < 0.1.

**Table 5:** The moderating effects of ICT adoption.

Var	(1)	(2)	(3)	(4)
	Income	Income	Risk Attitude	Risk Attitude
ICT		0.444 ***		0.287 ***
		(0.094)		(0.030)
MWE × ICT		−0.481 **		0.218 ***
		(0.198)		(0.064)
MWE	0.677 ***	0.854 ***	0.125 ***	−0.000
	(0.103)	(0.134)	(0.034)	(0.043)
Control	Yes	Yes	Yes	Yes
_cons	11.811 ***	11.504 ***	2.193 ***	1.980 ***
	(0.288)	(0.294)	(0.095)	(0.095)
Obs.	2863	2863	2863	2863
R-sqr	0.073	0.065	0.102	0.153

Note: Robust errors are in parenthesis, *** *p* < 0.01, ** *p* < 0.05.

**Table 6:** Direct Effects and Mediating Effects on Weak ties in Social Capital.

Var	(1)	(2)	(3)	(4)	(5)	(6)	(7)
	Weak Ties	Income	Weak Ties	Risk	Weak Ties	ICT	Weak Ties
Income			0.010 ***				
			(0.002)				
Risk					0.060 ***		
					(0.007)		
ICT							0.189 ***
							(0.009)
MWE	0.043 ***	0.677 ***	0.037 ***	0.125 ***	0.036 ***	0.092 ***	0.026 **
	(0.012)	(0.103)	(0.012)	(0.034)	(0.012)	(0.023)	(0.011)
Control	Yes	Yes	Yes	Yes	Yes	Yes	Yes
_cons	0.619 ***	11.811 ***	0.505 ***	2.193 ***	0.487 ***	0.720 ***	0.483 ***
	(0.034)	(0.288)	(0.043)	(0.095)	(0.036)	(0.064)	(0.032)
Obs.	2863	2863	2863	2863	2863	2863	2863
Sobel Tests		0.007 ***		0.008 ***		0.017 ***	
		(0.002)		(0.002)		(0.004)	
Total effect mediated		15%		17%		40%	

Note: Robust errors are in parenthesis, *** *p* < 0.01, ** *p* < 0.05.

**Table 7:** Direct Effects of MWE-year on Social Capital and Weak ties in Social Capital.

Var	(1)	(2)
	Social Capital	Weak Ties in Social Capital
MWE-year	0.348 ***	0.001 ***
	(0.053)	(0.001)
Control	Yes	Yes
_cons	35.804 ***	0.638 ***
	(0.095)	(0.034)
Obs.	2863	2863
R-sqr	0.035	0.153

Note: Robust errors are in parenthesis, *** *p* < 0.01.

**Table 8:** Results of balance test.

Sample	Ps R2	LR chi2	*p* > chi2	MeanBias	MedBias
Before matching	0.216	772.08	0.000	38.2	49.4
The nearest neighbor matching	0.009	14.03	0.121	5.2	5.7
Caliper matching (0.03)	0.004	10.98	0.277	3.5	3.3
Kernel matching (default 0.06 bandwidth)	0.009	14.03	0.121	5.2	5.7
Local linear regression matching	0.009	14.03	0.121	5.2	5.7

**Table 9:** The impact of MWE on social capital: PSM model results.

Method	Social Capital		
ATT	ATT	St. Dev	T Value
The nearest neighbor matching	9.483 **	3.864	2.453
Caliper matching (0.03)	9.461 **	4.531	2.097
Kernel matching (default 0.06 bandwidth)	9.483 **	3.863	2.452
Local linear regression matching	9.767 **	3.863	2.537

Note: Standard errors are in parenthesis, ** *p* < 0.05.

## Data Availability

The data that support the findings of this study were obtained from the survey conducted by the National Agricultural and Rural Development Research Institute at China Agricultural University.
